# The Alternative Sigma Factor RpoE2 Is Involved in the Stress Response to Hypochlorite and *in vivo* Survival of *Haemophilus influenzae*

**DOI:** 10.3389/fmicb.2021.637213

**Published:** 2021-02-12

**Authors:** Marufa Nasreen, Aidan Fletcher, Jennifer Hosmer, Qifeng Zhong, Ama-Tawiah Essilfie, Alastair G. McEwan, Ulrike Kappler

**Affiliations:** ^1^Australian Infectious Diseases Research Centre, School of Chemistry and Molecular Biosciences, The University of Queensland, St. Lucia, QLD, Australia; ^2^QIMR Berghofer Medical Research Institute, Herston, QLD, Australia

**Keywords:** extracytoplasmic function sigma factor, *H. influenzae*, hypochlorite, gene regulation, stress response

## Abstract

Extracytoplasmic function (ECF) sigma factors underpin the ability of bacteria to adapt to changing environmental conditions, a process that is particularly relevant in human pathogens that inhabit niches where human immune cells contribute to high levels of extracellular stress. Here, we have characterized the previously unstudied RpoE2 ECF sigma factor from the human respiratory pathogen *H. influenzae* (Hi) and its role in hypochlorite-induced stress. Exposure of *H. influenzae* to oxidative stress (HOCl, H_2_O_2_) increased *rpoE2* gene expression, and the activity of RpoE2 was controlled by a cytoplasmic 67-aa anti-sigma factor, HrsE. RpoE2 regulated the expression of the periplasmic MsrAB peptide methionine sulfoxide reductase that, in *H. influenzae*, is required for HOCl resistance, thus linking RpoE2 to HOCl stress. Interestingly, a HiΔ*rpoE2* strain had wild-type levels of resistance to oxidative stress *in vitro*, but HiΔ*rpoE2* survival was reduced 26-fold in a mouse model of lung infection, demonstrating the relevance of this sigma factor for *H. influenzae* pathogenesis. The HiRpoE2 system has some similarity to the ECF sigma factors described in *Streptomyces* and *Neisseria* sp. that also control the expression of *msr* genes. However, HiRpoE2 regulation extended to genes encoding other periplasmic damage repair proteins, an operon containing a DoxX-like protein, and also included selected OxyR-controlled genes. Based on our results, we propose that the highly conserved HiRpoE2 sigma factor is a key regulator of *H. influenzae* responses to oxidative damage in the cell envelope region that controls a variety of target genes required for survival in the host.

## Introduction

Extracytoplasmic function (ECF) sigma factors are one of the major signal transduction systems in bacteria and enable responses to a variety of environmental stimuli (Helmann, 2002; Staroń et al., 2009). Genes encoding ECF sigma factors are found in the majority of bacterial genomes, and at least 94 phylogenetically distinct groups of ECF sigma factors are known at present (Staroń et al., 2009; Pinto and Mascher, 2016). A distinguishing trait of ECF sigma factors is that their activity is inhibited by a so-called anti-sigma factor (ASF), which sequesters the ECF sigma factor until activation occurs through a variety of mechanisms (Mascher, 2013).

While ECF sigma factors are often highly abundant in versatile bacteria that can inhabit a wide range of environments, host-adapted bacteria tend to contain fewer of these alternative sigma factors. This is the case in the human respiratory pathobiont *H. influenzae* where only two ECF sigma factor-encoding genes are present. *H. influenzae* is a typical commensal bacterium of the nasopharyngeal microbiome, but it is also a major causative agent of acute respiratory tract infections such as otitis media, sinusitis, and pneumonia (Pettigrew et al., 2012; Slack, 2015). Additionally, *H. influenzae* is an early airway colonizer in cystic fibrosis and exacerbates several chronic respiratory tract diseases such as chronic obstructive pulmonary disease, bronchiectasis, and asthma (Essilfie et al., 2012; King, 2012; Finney et al., 2014; Van Eldere et al., 2014; King and Sharma, 2015). Current clinical isolates of *H. influenzae* are predominantly unencapsulated, non-typeable strains (NTHi), for which an increase in disease severity has been reported (Soeters et al., 2018). These traits, in combination with the increasing antibiotic resistance of isolate strains, have led to *H. influenzae* being classified as a WHO priority pathogen (Tristram et al., 2007; Macneil et al., 2011; King, 2012; Van Eldere et al., 2014; Wan Sai Cheong et al., 2015; World Health Organization., 2017; Soeters et al., 2018).

Clinical specimens of tissues affected by NTHi are characterized by high levels of host-induced oxidative stress and often show signs of hyperinflammation and tissue damage caused by the release of reactive chlorine or oxygen species (RCS and ROS, respectively), such as hypochlorite, superoxide, and hydrogen peroxide (Harrison et al., 2012, 2015; King, 2012). Oxidative stress can cause significant damage to the bacterial cell envelope, lipids, proteins, and DNA, where specific repair mechanisms are required that, in many bacterial species, have been shown to be controlled by ECF sigma factors (Gangaiah et al., 2014; Park et al., 2019). However, the function of ECF sigma factors in *H. influenzae* has only received limited attention so far. It was shown that *H. influenzae* genomes encode an ECF sigma factor that is closely related to the well-studied, envelope stress-induced RpoE sigma factor from *Escherichia coli*, and this ECF sigma factor was required for *H. influenzae* survival during infection of a macrophage cell line (Craig et al., 2002).

In this study, we describe for the first time the function of a second *H. influenzae* ECF sigma factor, RpoE2, that is located in a gene region that also encodes a periplasmic peptide methionine sulfoxide reductase, MsrAB, which is required for resistance of *H. influenzae* to oxidative damage caused by hypochlorous acid (HOCl) both *in vitro* and during infection (Nasreen et al., 2020). We have characterized the HiRpoE2/ASF system, its role in oxidative stress resistance, and its contribution to the survival of *H. influenzae* in a mouse model of infection. We also compare HiRpoE2 to related systems from *Neisseria gonorrhoeae*, *Neisseria meningitidis*, and *Streptomyces coelicolor* that control the expression of *msr* genes (Paget et al., 1998; Gunesekere et al., 2006; Hopman et al., 2010).

## Material and Methods

### Bacterial Strains, Media, and Growth Conditions

The bacterial strains and plasmids used in this study are listed in Supplementary Tables 1, 2. *E. coli* strains were cultured at 37°C using Luria–Bertani (LB) broth (Ausubel, 2002) with shaking at 200 rpm or statically on LB agar (1.5%, *w*/*v*) plates. Where necessary, LB was supplemented with 100 μg/ml ampicillin, 50 μg/ml spectinomycin, 20 μg/ml kanamycin, and/or 30 μg/ml trimethoprim.

*H. influenzae* strains were cultured either in BBL^TM^ Brain Heart Infusion (BHI) broth (Becton Dickinson) supplemented with 10 μg/ml hemin and 10 μg/ml β-nicotinamide adenine dinucleotide (NAD) (Johnston, 2010) or in chemically defined media (CDM) (RPMI 1640) (Sigma-Aldrich, R6504-1L): 24 mM sodium bicarbonate, 786 μM uracil, 25 mM HEPES (pH 7.5), 10 mM glucose, 7.5 mM inosine, 1 mM sodium pyruvate, 10 μg/ml NAD, and 10 μg/ml hemin) under microaerobic (5% CO_2_ and 3.7% O_2_), aerobic (5% CO_2_), or anaerobic (5% CO_2_, no oxygen) conditions at 37°C, with shaking at 200 rpm for aerobic and microaerobic conditions (Coleman et al., 2003). Difco^TM^ BHI (Becton Dickinson) and CDM (1.5%, *w*/*v*) agar plates were incubated at 37°C with 5% CO_2_. Where appropriate, BHI and CDM were supplemented with 20 μg/ml kanamycin and/or 50 μg/ml spectinomycin.

### Growth of *H. influenzae* in the Presence of HOCl

Hi2019^WT^ and Hi2019*^Δ*rpoE2*^* cell material freshly grown on CDM agar plates was used to inoculate the CDM broth to OD_600_ = 0.05. HOCl (10–15% sodium hypochlorite; Sigma Aldrich) was added to cultures in increasing concentrations (0–1,000 μM), and 200 μl of each culture were immediately added to a round-bottom 96-well microtiter plate (Greiner Bio-One) in replicate (*n* = 3). The plates were incubated under microaerobic conditions (2.8% O_2_ and 5% CO_2_) at 37°C, 200 rpm in a Clariostar multimode plate reader (BMG Labtech). OD_600_ measurements were recorded every 15 min for 24 h.

### Bactericidal Assays

*H. influenzae* cell material freshly grown on supplemented brain heart infusion (sBHI) plates was resuspended to an OD_600μl_ of 1.0 in 1x sterile phosphate-buffered saline (PBS). Of the bacterial culture, 900 μl were then combined with 100 μl of a freshly prepared 10x stock of the test compound, incubated at room temperature with gentle orbital shaking for 1 h followed by immediate serial dilution (up to 10^–7^) in BHI, and plating on sBHI plates. Assays used 25–250 μM HOCl, 100–750 mM H_2_O_2_, 1–5 mM paraquat, or 1–7 μg/ml polymyxin. Serum-mediated killing assays were performed as previously published (Lichtenegger et al., 2017). Hi2019 was grown to mid-log phase (OD_600nm_ of 0.5–0.6) and then harvested (800 × *g*, 10 min, 20°C) and resuspended in 1x PBS supplemented with 0.5% bovine serum albumin (BSA), 5 mM MgCl_2_, and 1 mM CaCl_2_. 2 × 10^7^ bacteria/ml were incubated with 0–10% serum for 45 min at 37°C, followed by serial dilution.

### Biofilm Formation

Biofilm formation and bacterial survival in biofilms were tested as in Dhouib et al. (2016). Hi2019 cell material from a freshly grown CDM plate was used to inoculate 20 ml CDM to OD_600_ = 0.05, followed by incubation at 37°C, 200 rpm, until OD_600_ = 0.2–0.3 was reached. For anaerobic conditions, bacteria were grown in CDM in completely filled 10- or 50-ml tubes and incubated at 37°C, without shaking in a CO_2_ incubator. For the assay, all cultures were diluted to an OD_600_ of 0.05 and 100 μl/well were added to U-bottom 96-well microtiter plates (TechnoPlas). Plates were incubated statically at 37°C, 5% CO_2_, for 16 h. For anaerobic conditions, plates were incubated in an anaerobic jar using Anaerocult catalysts (Merck) to remove oxygen. Detection of biofilm formation used crystal violet staining, as in Schembri and Klemm (2001). Viable cells present in the biofilm were determined by incubating each well with 200 μl of 0.1 mg/ml proteinase K for 10 min to dissolve the biofilm, followed by serial dilution (up 10^–7^ in BHI) and plating on sBHI plates for CFU/well determination.

### Tissue Cell Infection Assays

16HBE14 human bronchial epithelial cells were used for adherence and invasion assays, essentially as described in Dhouib et al. (2016). In brief, 16HBE14 cells were infected with Hi2019 (MOI = 100:1, bacteria:tissue cells) for 4 or 24 h. To determine titers of total adherent bacteria (i.e., all bacteria associated extra- or intracellularly with 16HBE14 cells), infected tissue cell layers were washed several times with fresh medium to remove non-adherent bacteria, lysed using 1% saponin, and serial dilutions of the lysate were plated on sBHI agar plates. To determine titers of internalized bacteria, infected tissue cells were treated with gentamycin (50 μg/ml) in MEM for 1 h prior to the wash steps and lysis.

### Mouse Model of Lung Infection

Experimental animal procedures were carried out in strict accordance with the recommendations in the Qld Animal Care and Protection ACT (2001) and the Australian Code of Practice for the Care and Use of Animals for Scientific Purposes, eighth edition. The protocols were approved by the Animal Care and Ethics Committees of QIMR Berghofer and the University of Queensland (QIMR/050/19). BALB/c female mice (6–7 weeks old) were inoculated intranasally with 30 μl of a bacterial suspension containing 10^7^ CFUs, as described in Dhouib et al. (2016). Groups of five mice were euthanized and necropsied at 0, 6, 24, 48, and 72 h. To quantify bacterial recovery, lungs were aseptically removed, homogenized in 1 ml 1 × sterile PBS, and serially diluted in the same buffer, followed by plating of the dilutions on sBHI plates. Bronchoalveolar lavage fluid (BALF) was also collected and CFU/ml determined. CFUs per lung were calculated as in Essilfie et al. (2011, 2012), and Essilfie et al. (2015). BALF was also cytocentrifuged and stained with May–Grunwald–Giemsa reagent (Merck) as per the manufacturer’s protocol and total immune cell numbers enumerated.

### Molecular Biology Methods

Standard methods were used throughout (Ausubel, 2002). PCR purification and plasmid isolation used the Wizard SV Gel and PCR Clean-Up System (Promega) and either the Genejet (Thermo Fisher Scientific) or Purelink Quick (Invitrogen) Plasmid Miniprep Kit, respectively. Genomic DNA was isolated using the Genomic DNA mini kit (Life Technologies). All kits were used according to the manufacturers’ instructions. Restriction enzymes were from Invitrogen or NEB. ANZAT4 ligase Mastermix (Invitrogen) or T4 ligase (NEB) were used for cloning. Standard PCR reactions used Gotaq Green Mastermix (Promega), high-fidelity PCR amplifications used Phusion Flash Mastermix (Thermo). Genomic DNA (gDNA) and PCR products were visualized using agarose gels (1–2%, *w*/*v*) in 1x TAE buffer, 1x SYBR^®^ safe DNA gel stain (Invitrogen), and a 1-kb plus DNA ladder (Thermo). DNA quantification used a Nanodrop spectrophotometer (Thermo Fisher Scientific). Transformation of *E. coli* used the calcium chloride method (Hanahan et al., 1991).

### Construction of Protein Expression Plasmids

Plasmids for the expression of RpoE2 or RpoE2 and the HrsE ASF with an N-terminal hexa-histidine Tag on RpoE2 were constructed using pProex-HTb (Invitrogen). Genes encoding the protein(s) of interest were amplified by PCR (Supplementary Tables 1, 2) and inserted into the plasmid following digestion with the appropriate restriction enzymes. Positive clones were confirmed *via* PCR using a combination of gene- and plasmid-specific primers (Supplementary Table 2). Expression of the target proteins was verified using LB cultures grown at 37°C and 200 rpm to an OD_600nm_ of 0.6–0.8 before the addition of 0.1 mM IPTG and further incubation at 37°C and 200 rpm overnight. Protein expression was verified using SDS-PAGE, protein purification used a His-GraviTrap column (1 ml, Cytiva) according to the manufacturer’s instructions, and 50–500 mM imidazole was used to elute the overexpressed proteins.

### Construction of pMU2385 Reporter Gene Plasmids

Fusions of Hi2019 gene promoter regions to a promoterless *lacZ* gene used the pMU2385 plasmid (Praszkier et al., 1992). Regions (245–865 bp) directly upstream of the coding regions of the Hi2019 genes *rpoE2* (gene: C645_RS08430, 284 and 865 bp), *msrAB* (252 bp), and *dmt* (gene: C645_RS08420, 245 bp) were amplified and cloned into the *Eco*RI and *Bam*HI restriction sites of pMU2385. Insertion was confirmed by PCR and DNA sequencing.

### Construction of a Hi2019^Δ^*^*rpoE2*^* Strain

To create a *Bam*HI restriction site in the *rpoE2* coding sequence, a 1,571-bp and a 1,201-bp DNA fragment containing the Hi*rpoE2* gene and flanking up- and downstream DNA regions were amplified from Hi2019 gDNA (Supplementary Tables 1, 2), digested with *Sac*I/*Bam*HI and *Eco*RI/*Bam*HI, respectively, and cloned into the *Sac*I and *Eco*RI sites of pBluescriptII SK(+) (Stratagene) creating pBlue_Hi*rpoE2*. The kanamycin cassette from pUC4K (Vieira and Messing, 1982) was amplified using primers pUC4K_PCR_F and R, digested with *Bam*HI, and inserted into pBlue_Hi*rpoE2*, leading to the formation of pBlue_Hi*rpoE*2_IS_kan. pBlue_Hi*rpoE*2_IS_kan was linearized with *Sca*I and transformed into Hi2019^WT^ using the protocol of Poje and Redfield (2003), M-IV solution and *H. influenzae* grown on BHI to an OD_600_ of 0.2–0.4. Cultures were pelleted and washed in 20 ml pre-warmed M-IV solution [described in Poje and Redfield (2003)]. Cell pellets were resuspended in 5 ml pre-warmed M-IV solution and incubated at 37°C for 100 min with 90 rpm shaking to develop competence. 1 μg of linearized plasmid was added to 500 μl of competent cells and incubated at 37°C, 5% CO_2_, for 30 min. Two milliliters of BHI broth was added to each transformation reaction, followed by another hour of incubation at 37°C, 5% CO_2_. The cells were washed once in BHI, resuspended in 1 ml BHI, plated on selective media, and incubated in a CO_2_ incubator at 37°C for 16–24 h. Colonies were screened by PCR to identify strains carrying the gene knockout mutation.

### RNA Isolation and cDNA Synthesis

*H. influenzae* strains were grown under aerobic, microaerobic, and anaerobic conditions in CDM glucose to OD_600_ ∼0.6 (aerobic and microaerobic) or to OD_600_ ∼0.4 for the anaerobic conditions. Two milliliters of the culture was added to 2 ml of RNA Protect Bacteria reagent (Qiagen), mixed by inversion, and incubated at room temperature for 5 min. Preserved bacterial cells were pelleted at 22,369 × *g* for 5 min and stored at −80°C for up to a week. Collection of RNA samples after exposure to oxidative stress reagents (200 μM HOCl, 5 mM paraquat, and 150 μM H_2_O_2_) used microaerobic cultures (200 ml medium/250 ml flask, 200 rpm) where the untreated sample (*t* = 0) was collected immediately before the addition of treatment and the additional samples after 30, 60, and 120 min of further incubation.

RNA was isolated using the Illustra RNAspin Mini kit (Cytiva), followed by gDNA removal (Turbo DNA Free Kit; Life Technologies) and cleanup of the reactions using the RNAspin Mini kit. RNA was quantified using a Qubit fluorimeter (Life Technologies). gDNA removal was assessed using PCR. cDNA was synthesized from 500 ng gDNA-free RNA using Superscript IV reverse transcriptase (Life Technologies).

### Quantitative RT-PCR

Quantitative reverse transcription PCR (qRT-PCR) was performed in 384-well plates as described in Othman et al. (2014) and Dhouib et al. (2016). Each reaction (10 μl) contained 2 μl of diluted cDNA (typically 1:100), 5 μl Quantinova SYBR green qPCR Mastermix (Qiagen), and primers at optimized concentrations between 0.5 and 2 μM (Supplementary Table 2). The cycle threshold (CT) values for all samples were determined using QuantstudioTM software version 1.3 (Thermo Fisher Scientific). PCR efficiencies were determined using LinReg Software version 2016.0 (Ruijter et al., 2009). The *gyrA* gene was used as the reference gene for relative quantification.

### Biochemical Methods

Standard methods were used throughout (Coligan, 2003; Ausubel et al., 2005). Protein concentrations were determined using the BCA-1 kit (Sigma-Aldrich). SDS-PAGE was performed as in Laemmli (1970) using a Low Molecular Weight Calibration Kit for SDS electrophoresis (Cytiva).

### Beta-Galactosidase Assay

Reporter gene assays were performed as described in Miller (1972). In brief, 5 ml LB supplemented with ampicillin (100 μg/ml) and trimethoprim (30 μg/ml) were inoculated to OD_600_ of 0.05 from overnight cultures of *E. coli* JM109λpir carrying combinations of pMU2385/pProex Htb plasmids (Supplementary Table 3). Cultures were incubated at 37°C, 200 rpm, to an OD_600_ of 0.4–0.8, harvested (2,370 × rcf, 4°C, 10 min), and resuspended in an equal volume of 1x PBS. 150 μl of resuspended bacteria was added to an equal volume of Z-buffer (0.06 M Na_2_HPO_4_, 0.06 M NaH_2_PO_4_, 0.01 M KCl, 0.001 M MgSO_4_ × 7H_2_O, and 2.7 ml/l β-mercaptoethanol, pH 7.0) and solubilized using 10 μl 1% (*w*/*v*) SDS and 10 μl chloroform. The cultures were vortexed for 10 s and the reaction started by addition of 50 μl of *o*-nitrophenyl-β-D-galactoside (4 mg/ml in 0.1 M potassium phosphate buffer, pH 7.0) and incubation in a 30°C water bath. Reactions were stopped on appearance of a yellow color by the addition of 125 μl 1 M Na_2_CO_3_, cell debris removed by centrifugation, and OD_420_ measured using microtiter plates. Miller units were calculated as in Miller (1972).

### Bioinformatic and Phylogenetic Analyses

RpoE2 and HrsE sequences from different *H. influenzae* strains were accessed *via* the NCBI website [35 sequence (RpoE2) and 27 sequence (HrsE/ASF) variants/alleles found in 713 *H. influenzae* genomes]. The sequences were imported into the MEGA phylogenetic analysis software in Fasta format and aligned using Clustal Omega (Sievers et al., 2011) as incorporated into the MEGA7 software package (Kumar et al., 2016). Phylogenetic tress were created in MEGA7 based on this alignment using the Neighbor-Joining method and bootstrapping (500 cycles) for robustness testing.

*In silico* promoter analyses were carried out for the Hi2019 *msrAB*, *rpoE2*, and the Dmt transporter protein encoding genes using the Prodoric Virtual Footprint website^1^ (Münch et al., 2005). Promoter and transcription factor binding site predictions used a 250-nt gene region from −200 to +50 of the selected genes and were obtained from the Hi2019 genome (acc. no. NZ_CP008740.1). Binding motifs found in the Virtual Footprint database (Münch et al., 2005) were used to test for the presence of sigma factor and transcription factor binding consensus sequences.

### Statistical Analyses

All data were analyzed using Microsoft Excel (2016; Microsoft, WA) and GraphPad Prism (8.2.1; GraphPad Software Inc., CA, United States). Two-tailed *t* tests were used to compare the mean CFU in the lung and in BALF at each time point. All other analyses used two-way ANOVA wherever possible. Šidák’s multiple comparisons test was used where applicable. A *p* equal to or <0.05 was considered statistically significant.

## Results

### The *H. influenzae* ECF Sigma Factor RpoE2 Controls Expression of Genes Encoding Proteins Essential for Oxidative Stress-Dependent Protein Repair

**FIGURE 1:**
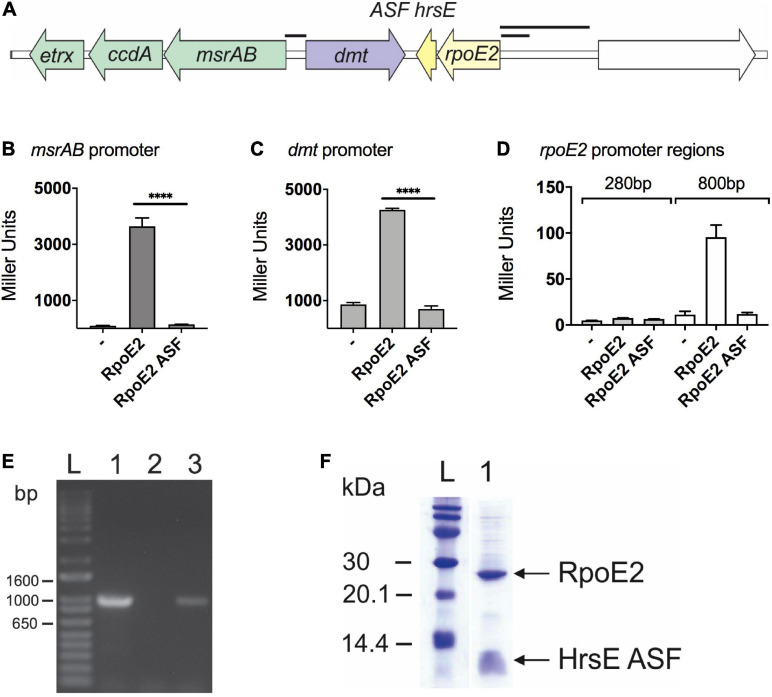
RpoE2 and its cognate anti-sigma factor (ASF), HseR, control the expression of neighboring genes encoding a peptide methionine sulfoxide reductase (*msrAB*) and a dmt-type transporter (*dmt*). **(A)** Schematic overview of the Hi2019 *rpoE2*-encoding gene region. *Black bars* indicate the promoter regions used in the reporter gene assays shown in other panels. **(B–D)** Activities of *H. influenzae msrAB* **(B)**, *dmt* **(C)**, and *rpoE2* **(D)** promoter regions in the *lacZ* reporter gene assays in *Escherichia coli* Jm109. Activity was tested in the presence of RpoE2 (label: *RpoE2*) and RpoE2 co-expressed with the HrsE ASF (label: *RpoE2-ASF*) and in the absence of specific inducer proteins (label: –). **(E)** Co-transcription analysis showing that, in *H. influenzae*, the genes encoding *rpoE2* and *hrsE* are co-transcribed. *Lane 1*, positive control, gDNA; *lane 2*, negative control, no template; l*ane 3*, Hi2019 cDNA. **(F)** Co-purification of recombinant 6xHis-HiRpoE2 and the HrsE ASF after heterologous expression in *E. coli*. ****p < 0.0001.

In the *H. influenzae* strain 2019 (Campagnari et al., 1987), the *rpoE2* gene encoding the second *H. influenzae* ECF sigma factor is located in the same gene region as the *msrAB* operon that encodes the HOCl-inducible MsrAB peptide methionine sulfoxide reductase (Nasreen et al., 2020; Figure 1A). A gene encoding a putative DMT family (drug/metabolite) transporter (“*dmt*,” C645_RS08420, COG0697) separates the *msrAB* operon from *rpoE2* and a gene encoding a small (67-amino acids, aa) putative cytoplasmic protein (Figure 1A). To test whether HiRpoE2 controls expression of *msrAB* and other genes present in this gene region, we fused the promoter regions (245–865 bp) for *msrAB*, *rpoE2*, and the *dmt* gene to the promoterless *lacZ* gene in pMU2385 (Praszkier et al., 1992) and tested the ability of HiRpoE2 to induce beta-galactosidase activity using *E. coli* JM109 as a host. The controls used pMU2385 and pProex-Htb plasmids without inserts (Figure 1 and Supplementary Figure 1).

The Hi*msrAB* promoter was strongly activated in the presence of RpoE2 (3,640 ± 302 Miller units, MU), but showed low residual activity (95 ± 11 MU) already in the absence of HiRpoE2 (Figure 1B). The *dmt* promoter also showed high activity in the presence of RpoE2 (4,264 ± 11 MU), but combined with a strong, RpoE2-independent activity (863 ± 70 MU) that was 9-fold higher than for the *msrAB* promoter (Figure 1C). The RpoE2-independent induction of the *msrAB* and *dmt* promoters indicates the presence of a second promoter in the upstream region of both genes.

To test the ability of HiRpoE2 to control the promoter of the *rpoE2* gene, we used both a 284-bp and an extended 865-bp region upstream of *rpoE2* that covers the large intergenic region upstream of *rpoE2* (Figures 1A,D). Only baseline activity was observed for the 284-bp *rpoE2* promoter (7.7 ± 0.1 MU), but a slight RpoE2-dependent activation was observed for the 865-bp promoter region (control = 11.4 ± 3.5 MU, RpoE2 = 95 ± 13 MU), indicating a very limited ability of RpoE2 to control the expression of the gene that encodes it (Figure 1D). We also demonstrated that the small gene that encodes a 67-aa protein is co-transcribed with Hi*rpoE2*, indicating a possible functional link (Figure 1E).

Together, these data clearly demonstrate that HiRpoE2 controls the expression of Hi*msrAB*, and since we have previously shown that *msrAB* is involved in the resistance of *H. influenzae* to HOCl, it suggested that RpoE2 mediates a response to oxidative stress or HOCl in *H. influenzae*.

### The *msrAB–rpoE2* Gene Region Is Highly Conserved in *H. influenzae* Genomes

**FIGURE 2:**
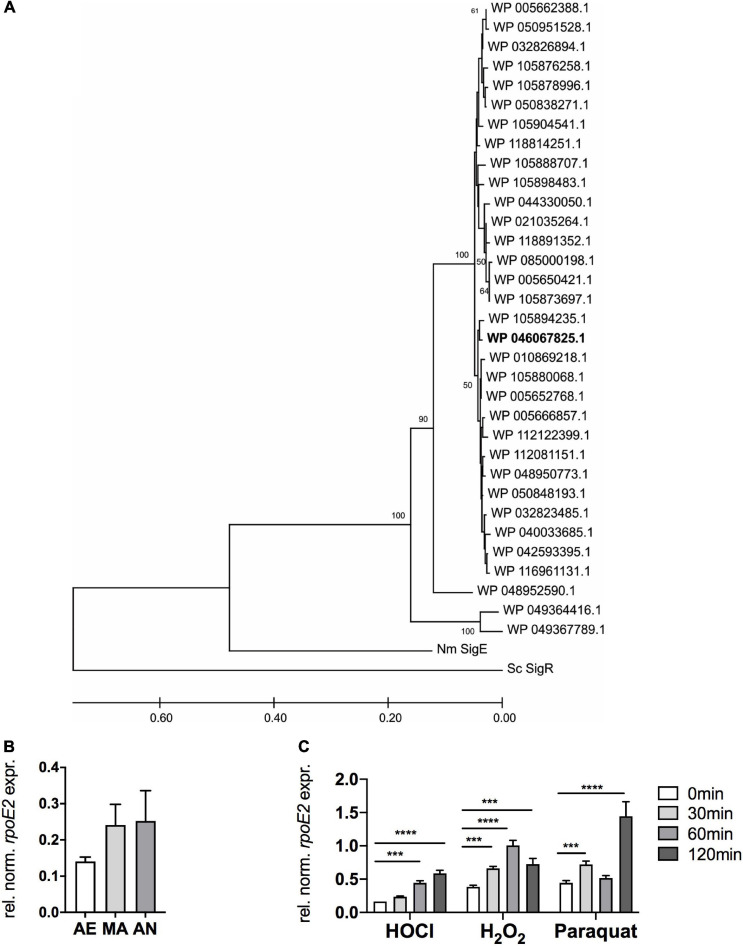
RpoE2 is highly conserved in *H. influenzae* strains and *rpoE2* gene expression increases following exposure to oxidative stress. **(A)** Phylogenetic relationships between the RpoE2 sequence variants found in *H. influenzae* strains. The phylogenetic tree was created using the neighbor-joining method with robustness testing using 500 bootstrap cycles. The majority of the HiRpoE2 protein sequences formed two related clades, while the remaining 10% formed a small cluster of slightly more divergent sequences (96% sequence identity to the main cluster). Three sequence types (WP_005650421.1, *n* = 153; WP_005666857.1, *n* = 75; and WP_005662388.1, *n* = 175) accounted for 403 or 56.5% of the sequences analyzed. The Hi2019 RpoE2 belongs to the WP_046067825.1 sequence type (in bold). HiRpoE2-related sequences from *Neisseria meningitidis* Nm3682 (“Nm SigE”, AIZ23068.1) and *Streptomyces coelicolor* A3(2) SigR (“Sc SigR”, CAB94601.1) were used as outgroups. **(B)** Expression of Hi*rpoE2* under aerobic (*AE*), microaerobic (*MA*), and anaerobic (AN) conditions. **(C)** Effects of hypochlorous acid (HOCl, 200 μM), H_2_O_2_ (150 μM), and paraquat (5 mM) on expression of Hi*rpoE2* over time. Gene expression data from qRT-PCR **(B,C)** are reported as relative expressions after normalization to the expression of the *H. influenzae gyrA* gene. Statistical testing used two-way ANOVA. ****p < 0.0001, ***p < 0.001.

Analysis of 713 publicly available *H. influenzae* genomes revealed that both the *rpoE2* gene and the gene encoding a small putative ASF protein are completely conserved in all strains. Further analyses identified 33 HiRpoE2 protein sequence variants with an average of 93% sequence identity. Thirty of these sequence variants (97% average sequence identity) formed two closely related clades, with three sequences being more divergent [85% (*n* = 1) and 75% (*n* = 2) sequence identity] (Figure 2A). The presence of small cytoplasmic ASFs is a typical feature of group 13 ECF sigma factors, to which HiRpoE2 belongs, and our analyses revealed the existence of 27 HiRpoE2 ASF sequence variants that also formed two closely related groups with three additional divergent sequence types (Supplementary Figure 2). The three more divergent sequence variants identified for RpoE2 and the putative ASF belong to a group of *H. influenzae* strains identified in the same study (Roach et al., 2015) and that show only 67 and 39.7% symmetrical identity to the most closely related *H. influenzae* genomes, explaining the relatively larger divergence of their RpoE2 and ASF sequences. In fact, the most divergent sequences (RpoE2: WP_049364416.1, ASF: WP_049364417.1) also occur in two strains of *Haemophilus parainfluenzae*.

The high conservation extended to the *rpoE2* gene region, which in the majority of strains was identical to the one found in Hi2019 (Figure 1A). However, variations occurred in a minority of *H. influenzae* strains (representatives of RpoE2 sequence types WP_005693910.1, WP_005662386.1, WP_044334451.1, and WP_048949909.1), where three genes, excluding a *dmt* transporter-encoding gene, separated the *msrAB* and *rpoE2* operons. In two additional groups of strains (WP_049367789.1 and WP_049364416.1, both of them representing the divergent RpoE2 sequence types), the *msrAB* operon was located at a distant chromosomal location.

Overall, the high sequence identity of HiRpoE2 and ASF proteins and their presence in the *H. influenzae* core genome indicate strong functional conservation.

### The HiRpoE2 ASF Is Related to ASFs From *Neisseria* and *Streptomyces* sp. and Controls the Activity of HiRpoE2

To determine whether the putative *H. influenzae* ASF can control RpoE2 activity, we then carried out a functional evaluation of the ASF protein using a protein expression plasmid, pProex-HiRpoE2-ASF, designed to express an N-terminally His-tagged RpoE2 protein and an untagged ASF protein. Following protein expression using this plasmid, the two proteins co-purified (Figure 1F), and the identity of the co-purifying small protein was confirmed by protein sequencing (Supplementary Table 4). Reporter gene assays then showed that the presence of the putative ASF abolished the activation of the *msrAB* promoter by RpoE2, confirming that this 67-aa protein is indeed an effective RpoE2-ASF, and we have therefore named it HrsE (*Haemophilus* Regulator of Sigma E) (Figure 1B). Reporter gene assays also showed that the observed activation of both the *dmt* and *rpoE2* promoters was reversible in the presence of HiHrsE (Figures 1C,D).

The HiHrsE ASF shares similarity with two characterized peptide ASFs from *N. meningitidis* and *S. coelicolor* that are also associated with ECF group 13 sigma factors (Kang et al., 1999; Hopman et al., 2010; Kallifidas et al., 2010). The two characterized sequences contain a so-called ZAS motif (C/H–X_23__–__26_–H–X_3_–C–X_2_–C) that mediates the binding of a functionally relevant zinc ion (Newman et al., 2001; Paget et al., 2001; Ilbert et al., 2006; Hopman et al., 2010), and these residues are conserved in HiHrsE (Supplementary Figure 3).

### Oxidative Stress Increases Expression of Hi*rpoE2*

Given its potential role in regulating the response to oxidative stress, we then tested the expression of Hi*rpoE2* in the presence and absence of H_2_O_2_, HOCl, and paraquat using qRT-PCR. We also investigated the expression of Hi*rpoE2* in response to different oxygen tensions that the bacteria may encounter during infection and host colonization.

In the absence of oxidative stress treatments, *rpoE2* expression increased moderately when the oxygen concentrations decreased (Figure 2B). Low oxygen tensions are typical at sites of infection and in biofilms.

Following treatment with 200 μM HOCl, *rpoE2* expression increased steadily over the 120 min of experimental time (3.6-fold maximum induction) (Figure 2C), while after H_2_O_2_ treatment (150 μM), *rpoE2* expression peaked at 60 min post-exposure (2.6-fold induction) and then started to decrease. In contrast, paraquat exposure (5 mM) resulted in a bimodal expression pattern with a small increase of expression after 30 min (1.6-fold) and a large increase after 120 min (3.1-fold). This second expression peak may not be associated with the ROS generated by the initial paraquat exposure, but may reflect a secondary response to derivative oxidizing compounds. While HOCl and H_2_O_2_ both triggered increases in *rpoE2* expression, the responses were relatively slow, taking at least 60 min to reach peak expression (Figure 2C). This is not consistent with the strong 2.5-fold increase in *msrAB* gene expression observed within 30 min of treatment with HOCl (Nasreen et al., 2020), which indicates that *de novo* RpoE2 synthesis is likely a secondary response to ROS or RCS exposure and that RpoE2 molecules already present in *H. influenzae* mediate the initial fast response to HOCl and, possibly, H_2_O_2_ treatment. The different expression patterns of *msrAB* and *rpoE2* also suggest that there may be differences in the types of oxidizing agents that inactivate the HiHrsE ASF compared to those that trigger *rpoE2* gene expression.

### Loss of RpoE2 Does Not Significantly Increase the Sensitivity of *H. influenzae* to Oxidative Stress

**FIGURE 3:**
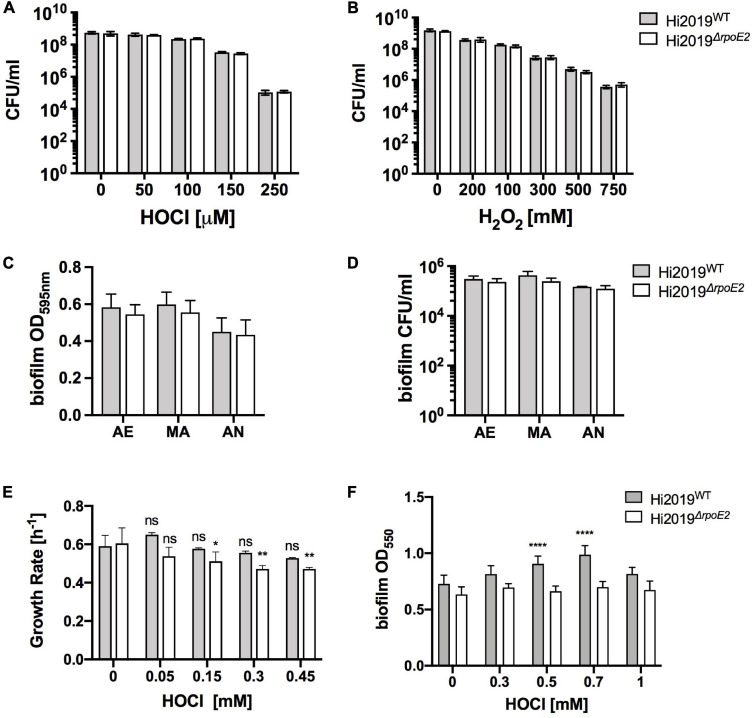
A Hi2019^Δ^*^*rpoE2*^* strain showed no increase in oxidative stress sensitivity and only slight changes in growth and biofilm formation in the presence of HOCl. **(A,B)** Killing of Hi2019^WT^ and Hi2019^Δ^*^*rpoE2*^* by exposure to increasing concentrations of HOCl **(A)** and H_2_O_2_ **(B)**. **(C,D)** Biofilm formation **(C)** and biofilm survival **(D)** of Hi2019^WT^ and Hi2019^Δ^*^*rpoE2*^* under microaerobic and anaerobic conditions in the absence of oxidative stress. **(E)** Growth rates of Hi2019^WT^ and Hi2019^Δ^*^*rpoE2*^* in the presence of increasing amounts of hypochlorous acid (HOCl). **(F)** Biofilm formation under microaerobic conditions of Hi2019^WT^ and Hi2019^Δ^*^*rpoE2*^* in the presence of increasing amounts of HOCl. *Gray bars*, Hi2019^WT^; *white bars*, Hi2019^Δ^*^*rpoE2*^*. Statistical analyses (two-way ANOVA, strain–time or strain–conc.) returned non-significant changes for the comparisons between Hi2019^WT^ and Hi2019^Δ^*^*rpoE2*^* at the same time point or concentration for panels **(A–D)**. For panels **(E,F)** all Hi2019^WT^ or Hi2019^Δ^*^*rpoE2*^* values were compared to the control (no HOCl) sample for the same strain; values are shown *directly above each bar*. *p < 0.05, **p < 0.01, ****p < 0.0001.

To elucidate the role of RpoE2 in *H. influenzae* physiology, we constructed a Hi2019^Δ^*^*rpoE2*^* mutant strain and verified it using PCR. As a Hi2019^Δ^*^*msrAB*^* strain was sensitive to HOCl-mediated killing and RpoE2 controls the expression of *msrAB*, we expected to see a similar phenotype for the Hi2019^Δ^*^*rpoE2*^* strain. However, contrary to expectations, killing assays with HOCl, H_2_O_2_, or paraquat showed that Hi2019^Δ^*^*rpoE2*^* was not more susceptible to killing by either of these substances than Hi2019^WT^ (Figures 3A,B and Supplementary Figure 4).

In the absence of oxidative stress, biofilm formation and survival in the biofilm, physiological traits essential for *H. influenzae* virulence, were also unaffected by the *rpoE2* mutation (Figures 3C,D). However, in the presence of increasing amounts of HOCl (up to 1 mM), biofilm formation for Hi2019^WT^ increased slightly up to 0.7 mM HOCl (a 1.3-fold increase), while for Hi2019^Δ^*^*rpoE2*^* biofilm formation did not change throughout (Figure 3E). For microaerobic growth in the presence of HOCl, the growth rates for Hi2019^Δ^*^*rpoE2*^* consistently declined starting from 150 μM HOCl, while for the wild type (WT) no change in the growth rate was observed at concentrations up to 450 μM (Figure 3F). In summary, while the loss of RpoE2 did not lead to a change in susceptibility to ROS or RCS killing, there were subtle changes in the growth rates and biofilm formation that may have some relevance *in vivo*.

### Hi2019^Δ^*^*rpoE2*^* Showed Wild-Type Infection Levels of Cultured Tissue Cells, but Was Attenuated in a Mouse Model of Lung Infection

**FIGURE 4:**
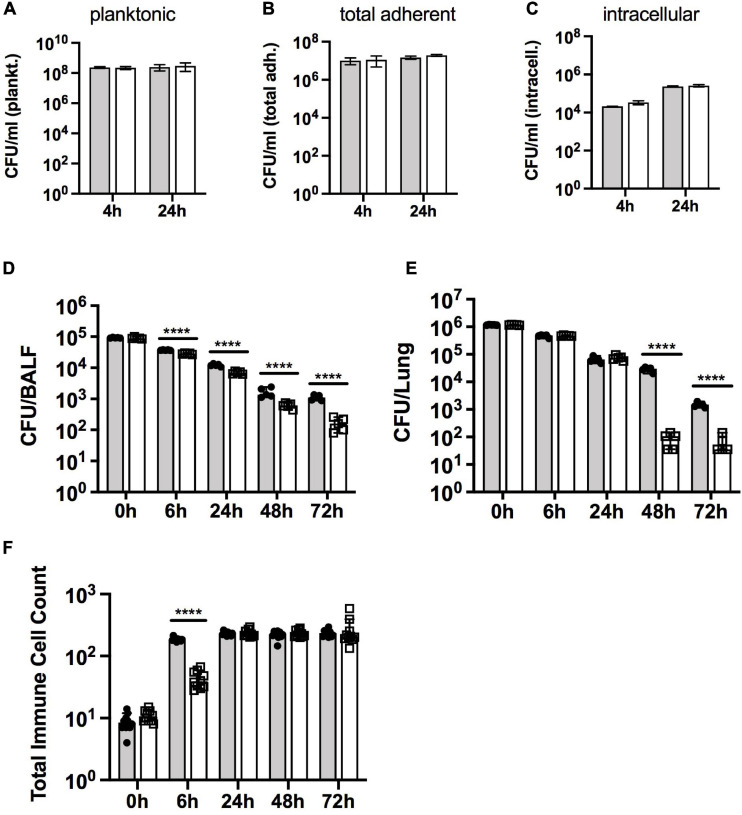
Survival of Hi2019^WT^ and Hi2019^Δ^*^*rpoE2*^* in different models of *H. influenzae* infection. **(A–C)** Infection of 16HBE14 tissue cells. Planktonic **(A)**, total adherent **(B)**, and intracellular **(C)** bacteria preferred notation – CFU/ml are shown after 4 and 24 h of infection. **(D–F)** Survival of Hi2019^WT^ and Hi2019^Δ*rpoE2*^ in a mouse model of lung infection. Bacterial cell numbers (in CFU) are shown per bronchoalveolar lavage fluid (BALF) **(D)** and lung tissue **(E)**. Giemsa staining was used to determine immune cell (neutrophil and macrophage) count changes over time in BALF **(F)**. *Gray bars*, Hi2019^WT^; *white bars*, Hi2019^Δ^*^*rpoE2*^*. Statistical analyses used Student’s *t* tests, comparing CFU for Hi2019^WT^ and Hi2019^Δ^*^*rpoE2*^* at each time point. ****p < 0.0001.

ROS and RCS such as HOCl are usually encountered during contact with host cells, so we tested the survival of the Hi2019^Δ^*^*rpoE2*^* strain in two different models of infection. During infection of 16HBE14 human bronchial epithelial cells, the Hi2019^Δ^*^*rpoE2*^* strain showed no difference in planktonic survival, adherence to, or invasion of the tissue cells compared to the wild type (*n* = 3 independent experiments) (Figures 4A–C). This was in contrast to our results for Hi2019^Δ^*^*msrAB*^*, where tissue cell invasion was impaired (Nasreen et al., 2020).

However, in a mouse model of lung infection, the survival of Hi2019^Δ^*^*rpoE2*^* was clearly attenuated compared to the Hi2019^WT^ strain. Bacterial loads in BALF steadily decreased compared to Hi2019^WT^, from a 1.3-fold reduction after 6 h to a 6.7-fold reduction after 72 h (all *p* < 0.0001, 2-way ANOVA) (Figures 4D,E). In contrast, the survival of the mutant strain in lung tissue was not affected during the first 24 h of the experiment, but a significant reduction in bacterial loads (26- and 4-fold, *p* < 0.0001, 2-way ANOVA) was observed after 48 and 72 h of infection (Figures 4D,E). During the experiment, the numbers of neutrophils and macrophages present in the lung tissue increased to similar levels for both *H. influenzae* strains, however, for Hi2019^Δ^*^*rpoE2*^*, the increase was somewhat delayed. While after 6 h of infection, the neutrophil and macrophage numbers in BALF had increased 21-fold for Hi2019^WT^ compared to the uninfected control; for Hi2019^Δ^*^*rpoE2*^* only a 4-fold increase (*p* < 0.0001, 2-way ANOVA) was seen (Figure 4F). This documents a role for RpoE2 in supporting *H. influenzae* virulence in a whole animal model of infection, despite the absence of a strong oxidative stress-related phenotype during the *in vitro* characterization of the mutant strain.

### HiRpoE2 Controls the Expression of Genes Involved in Methionine Sulfoxide Repair and Also Affects Expression of Oxidative Stress Defense Genes Controlled by OxyR

Despite the fact that RpoE2 is required for expression of MsrAB, which mediates *H. influenzae* resistance to HOCl stress (Nasreen et al., 2020), the Hi2019^Δ^*^*rpoE2*^* strain lacked a strong oxidative stress-related phenotype, and this prompted us to assess expression of genes involved in oxidative stress defense in Hi2019^Δ^*^*rpoE2*^*.

We included the RpoE2-regulated *msrAB* and *dmt* genes and a gene encoding another periplasmic methionine sulfoxide reductase, *mtsZ*, that we characterized earlier (Dhouib et al., 2016). Additionally, the *sodA* gene encoding superoxide dismutase (Kroll et al., 1993) and the OxyR-regulated genes encoding catalase (*hktE*), peroxiredoxin (*pgdX*), and the ferritin-like DPS protein (*dps*), all of which are induced by exposure of *H. influenzae* to H_2_O_2_ (Harrison et al., 2007; Whitby et al., 2012), were included.

**FIGURE 5:**
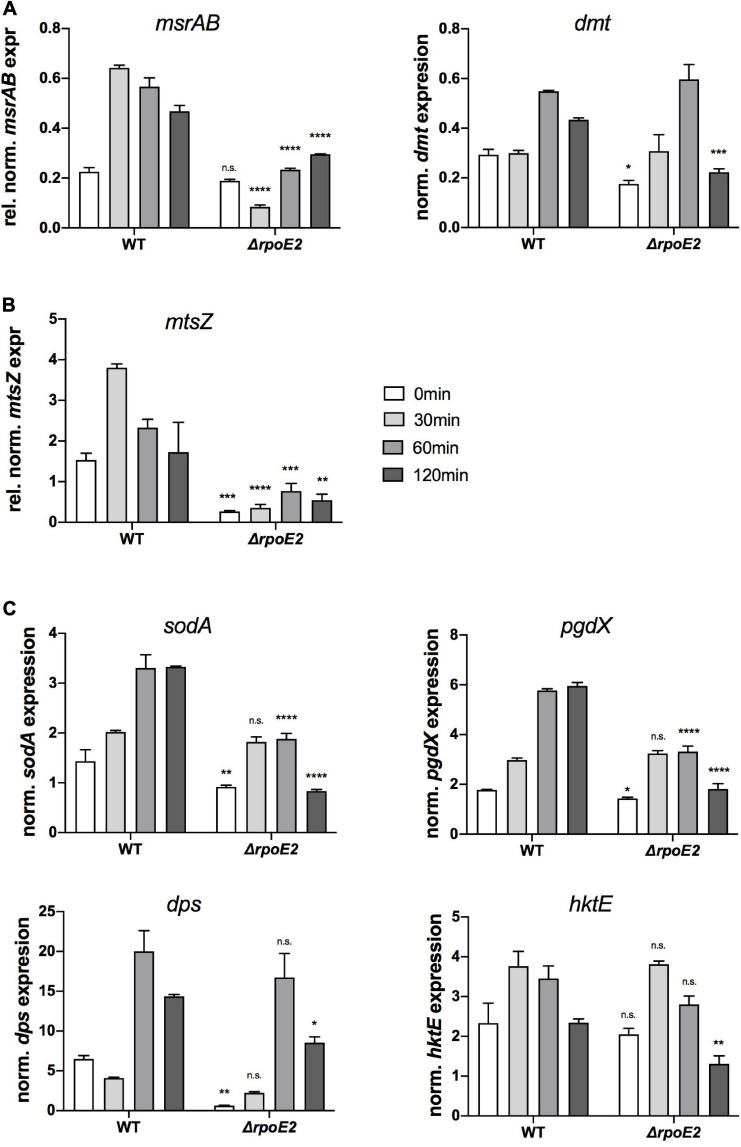
Expressions of putative RpoE2-regulated genes **(A,B)** and genes encoding proteins known to be involved in *H. influenzae* oxidative stress response **(C)** in Hi2019^WT^ and Hi2019^Δ^*^*rpoE2*^* following exposure to 200 μM HOCl. **(A)** Expressions of the RpoE2-regulated *msrAB* and *dmt* genes. **(B)** Expression of a gene (*mtsZ*) encoding MtsZ, a periplasmic Mo-containing methionine sulfoxide reductase. **(C)** Expressions of genes encoding superoxide dismutase (*sodA*), peroxiredoxin (*pgdX*), catalase (*hktE*), and the ferritin-like protein Dps (*dps*). *hktE*, *dps*, and *pgdX* are part of the *H. influenzae* OxyR regulon. Gene expression data generated by qRT-PCR are reported as relative expressions after normalization to the expression of the *gyrA* gene. Statistical testing of expression changes relative to Hi2019 WT used two-way ANOVA. Statistically significant changes are indicated *above the bars* representing the gene expression of Hi2019^Δ^*^*rpoE2*^*. *p < 0.05, **p < 0.01, ***p < 0.001, ****p < 0.0001; *n.s.*, not significant.

As shown previously (Nasreen et al., 2020), expression of *msrAB* was strongly induced within 30 min of HOCl exposure in Hi2019^WT^, while in Hi2019^Δ^*^*rpoE2*^* after 30 min of HOCl treatment, *msrAB* expression was actually reduced 2-fold, while expression at 60 and 120 min slightly increased compared to *t* = 0 min (Figure 5A). We propose that this residual expression of the *msrAB* gene might explain the lack of a HOCl-sensitivity phenotype in Hi2019^Δ^*^*rpoE2*^*, as it would allow for the production of a basal level of MsrAB protein. Expression of the *dmt* gene that is located between the *msrAB* and *rpoE2* operons was significantly lower in Hi2019^Δ^*^*rpoE2*^* compared to Hi2019^WT^ prior to HOCl treatment (1.6-fold reduction, *p* = 0.03, 2-way ANOVA), but showed HOCl-dependent induction with peak expression reached after 60 min in both strains (Figure 5A). We attribute this expression pattern to the second, RpoE2-independent promoter identified as part of the reporter gene assays.

Interestingly, expression of the *mtsZ* gene that encodes an enzyme that reduces free MetSO to Met (Dhouib et al., 2016) showed a similar pattern to *msrAB*, with strong induction within 30 min in Hi2019^WT^ and only residual expression in the Hi2019^Δ^*^*rpoE2*^* strain, suggesting that the expression of *mtsZ* is also controlled by RpoE2 (Figure 5B).

In contrast, expression of the four genes known to be part of the general oxidative stress defense in *H. influenzae* was much less affected by the loss of RpoE2 (Figure 5C). While a decrease in the basal gene expression levels prior to HOCl treatment was observed for *pgdX, sodA*, and *dps* (Figure 5C), expression levels of catalase (*hktE*) and *dps* were essentially unaltered in Hi2019^WT^ and Hi2019^Δ^*^*rpoE2*^* up to 60 min post-HOCl exposure, but showed a reduction at 120 min. The *sodA* and *pgdX* genes showed a statistically significant reduction in peak expression, which in these genes occurred after 60 and 120 min, respectively.

### HiRpoE2 Controls Expression of Genes Homologous to Members of the *N. meningitidis* SigE Regulon

**FIGURE 6:**
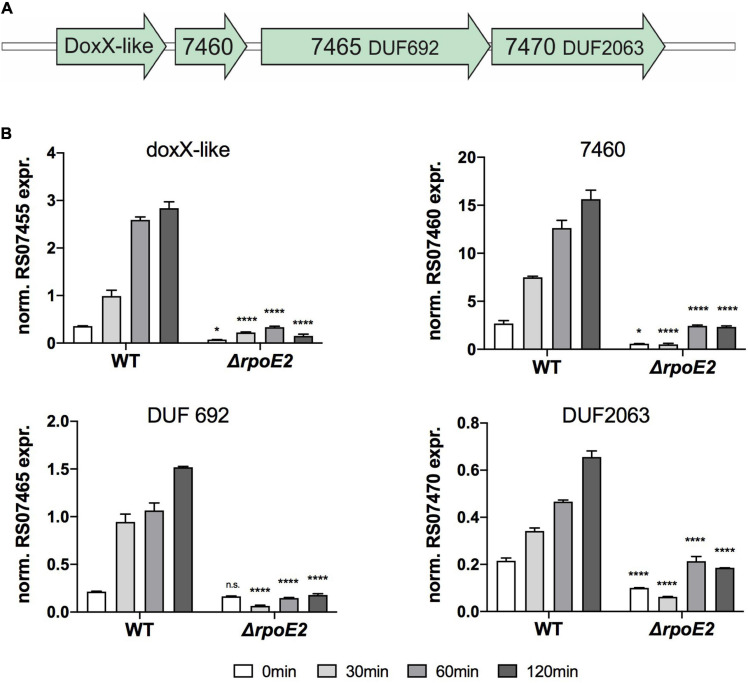
Expression of putative RpoE2-regulated genes encoding a DoxX-like protein and three putative accessory proteins. **(A)** gene cluster encoding the Hi2019 *doxX* gene region. **(B)** Expression of *H. influenzae* genes encoding a DoxX-like protein (RS07455) and three hypothetical proteins containing conserved domains of unknown function (*DUF*)—RS07460 (COG3767, periplasmic), RS07465 (COG3220/UPF0276, cytoplasmic), and RS07470 (COG3219, DUF2063, cytoplasmic)—in Hi2019^WT^ and Hi2019^Δ^*^*rpoE2*^* following exposure to 200 μM HOCl. Gene expression data are reported as relative expression after normalization to expression of the *gyrA* gene. Statistical testing of expression changes in Hi2019^Δ^*^*rpoE2*^* relative to Hi2019^WT^ used two-way ANOVA. Statistically significant changes are indicated *above the bars* representing the gene expression of Hi2019^Δ^*^*rpoE2*^*. *p < 0.05, **p < 0.01, ***p < 0.001, ****p < 0.0001; *n.s.*, not significant.

Our analyses so far indicate that the HiRpoE2 regulatory system resembles the SigE regulon from *N. meningitidis*, where the ECF sigma factor SigE controls expression of the Nm*trx*-*msrAB* gene, a *sigE* small RNA (sRNA) and 10 additional genes encoding outer membrane proteins, a NirK-like protein, the SigE and ASF proteins, and a cluster of three hypothetical proteins and a DoxX family protein (Gunesekere et al., 2006; Hopman et al., 2010). Analyses of the Hi2019 genome identified a gene cluster encoding proteins identical to the DoxX gene cluster from *Neisseria* (Hi2019 genes C645_RS07455–C645_Rs07470) (Figures 6A–D). These proteins showed significant homology to their *Neisseria* counterparts, while with the exception Hi*msrAB* and a gene encoding an opacity-associated outer membrane protein (Hi2019 gene C645_RS07000), no homologs of any of the other NmSigE target genes or the *Neisseria sigE* sRNA were identified in Hi2019.

Following exposure to HOCl, expression levels of the DoxX cluster genes in Hi2019^WT^ and Hi2019^Δ^*^*rpoE2*^* varied, but all four genes showed a similar pattern of induction with increases starting 30 min post-exposure and highest expression levels reached after 120 min in Hi2019^WT^, a pattern reminiscent of that of the *rpoE2* gene. Interestingly, expression of all four genes was strongly RpoE2-dependent, and no significant induction of gene expression was observed in Hi2019^Δ^*^*rpoE2*^* following HOCl treatment (Figures 6A–D). This demonstrates that functional similarities exist between the HiRpoE2 and Neisseria SigE regulons, and the function of the proteins encoded by the *doxX* operon should be studied in detail in the future.

## Discussion

The RpoE2 ECF sigma factor is one of only two conserved ECF sigma factors in *H. influenzae*, and here we have shown that RpoE2 is an oxidative stress-responsive alternative sigma factor that regulates functions associated with HOCl resistance and envelope protein repair.

The activity of RpoE2 is controlled by a small peptide ASF, HrsE, which makes it distinct from the other highly conserved *H. influenzae* ECF sigma factor, RpoE, that is closely related to the well-studied *E. coli* SigE and, like its *E. coli* counterpart, is encoded by an operon that also contains genes encoding the membrane-bound RseAB ASF typical of group 1 ECF sigma factors (Craig et al., 2002).

The HiHrsE ASF resembles other ECF group 13 peptide ASFs, all of which share conserved residues involved in binding a zinc ion (ZAS motif) that are also conserved in HiHrsE, suggesting conservation of function between these systems.

HiRpoE2 is most closely related to the SigE ECF sigma factor from *Neisseria* species. In *Neisseria*, H_2_O_2_ was reported as an inducer of the SigE response, and mapping of the SigE regulon in *N. meningitidis* found that it consists of only 10 genes, including those encoding Nm*sigE* and its cognate ASF (Gunesekere et al., 2006; Hopman et al., 2010; Huis in ’T Veld et al., 2011). In contrast, here, we have shown that HiRpoE2 gene expression is induced in response to exposure to HOCl, H_2_O_2_, and paraquat, indicating a much wider role in *H. influenzae* oxidative stress defense and likely protection of *H. influenzae* from thiol stress.

The *Neisseria* SigE protein was found to be non-essential for host cell interactions in a tissue cell model of infection (Du et al., 2005), and we observed a similar phenotype for our Hi2019^Δ^*^*rpoE2*^* strain, as well as an absence of a strong ROS-sensitivity phenotype. However, in a mouse model of infection, Hi2019^Δ^*^*rpoE2*^* showed reduced fitness evidenced by faster clearance from both lung tissue and the airways, indicating a specific role for this sigma factor *in vivo*.

These observations may be linked to changes in the expression levels of the Hi*msrAB* gene, which is required for resistance of *H. influenzae* to HOCl (Nasreen et al., 2020). In addition, MsrAB was shown to have immunomodulatory effects (Nasreen et al., 2020), and the *in vivo* phenotype of Hi2019^Δ^*^*rpoE2*^* may be related to lower levels of *msrAB* expression that affect this function.

Our preliminary exploration of *H. influenzae* genes regulated by RpoE2 indicates that, in addition to *msrAB*, RpoE2 controls the expression of a conserved gene encoding a DMT-type transporter that is found in the *msrAB* gene region and another periplasmic MetSO reductase, MtsZ, as well as genes encoding proteins homologous to members of the *Neisseria* SigE regulon. Most of these proteins are functionally uncharacterized, but regulation of a gene encoding a DoxX-like protein by RpoE2 is significant, as a DoxX-like protein from *Mycobacterium tuberculosis* has been shown to form a membrane-bound thiol oxidoreductase complex with an Fe-containing superoxide dismutase and a sulfur transferase, SseA (Nambi et al., 2015). In *M. tuberculosis*, this protein complex maintains mycothiol homeostasis and is required for bacterial survival in mice and activated macrophages (Nambi et al., 2015). The *H. influenzae* DoxX-like membrane protein is encoded in a gene region that also contains genes encoding a periplasmic protein and two potentially cytoplasmic proteins, all of which belong to ‘domain of unknown function’ groups, and it is a distant relative of the DoxX protein characterized in *M. tuberculosis*.

**FIGURE 7:**
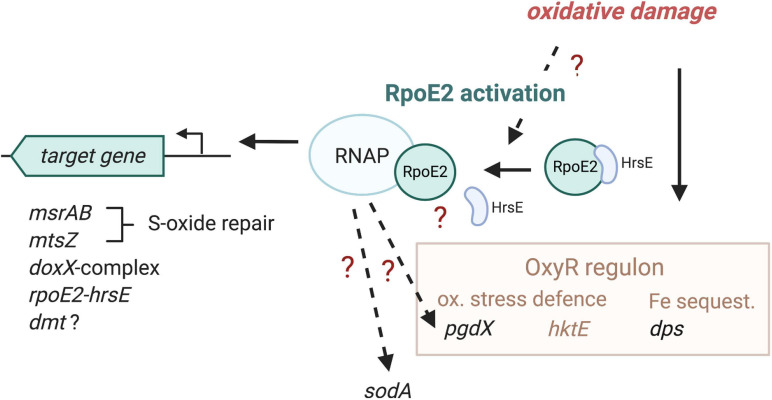
Proposed model of HiRpoE2 function. HiRpoE2 is activated following exposure of the *H. influenzae* cell to oxidative stress, which likely leads to a dissociation of the HrsE anti-sigma factor (ASF) from RpoE2, allowing RpoE2 to activate the expression of target genes, several of which, such as *msrAB* and *mtsZ*, mediate extracellular oxidative damage repair reactions. *Question marks* and *broken lines* denote processes that are not fully elucidated at the molecular level. *Gene names in black font*, expression affected by RpoE2; *brown* and *brown font*, OxyR-associated functions, including genes known to be regulated by OxyR.

Our data show that HiRpoE2 is a new element in the *H. influenzae* defense against oxidative stress, where OxyR and also ArcA have previously been identified as major regulators (Harrison et al., 2007; Wong et al., 2007; Whitby et al., 2012). *H. influenzae* OxyR controls the expression of key oxidative stress response enzymes such as catalase and peroxiredoxin, and both regulators contribute to iron sequestration *via* the expression of, e.g., the ferritin-like Dps protein (OxyR) and a Dps-like protein (ArcA) (Harrison et al., 2007; Wong et al., 2007; Whitby et al., 2012). Notably, both OxyR and ArcA appear to be primarily involved in controlling the expression of enzymes that protect the *H. influenzae* cytoplasm from oxidative stress, while RpoE2 directly regulates the expression of at least two periplasmic enzymes, MtsZ and MsrAB, both of which are involved in the repair of oxidative damage, and an uncharacterized DoxX-containing protein complex that also contains a periplasmic component. An interesting observation is that a certain overlap appears to exist between the OxyR regulon and the HiRpoE2 regulon, where *dps* and *pgdX* expression was altered in Hi2019^Δ^*^*rpoE2*^* (Figure 7). Our data also indicate that there are additional levels of complexity in RpoE2-based gene regulation as the induction patterns of RpoE2-dependent genes varied, and bioinformatic analyses also identified potential OxyR binding sites upstream of *rpoE2*, *msrAB*, and *dmt*.

In summary, we propose that the strictly conserved HiRpoE2 sigma factor is required for maintaining the integrity of *H. influenzae* outer membrane, periplasmic and possible membrane proteins and controls key elements of the *H. influenzae* cell envelope oxidative stress defense.

## Data Availability Statement

The original contributions presented in the study are included in the article/Supplementary Material, further inquiries can be directed to the corresponding author.

## Ethics Statement

The animal study protocols were approved by Animal Care and Ethics Committees of QIMR Berghofer and the University of Queensland (QIMR/050/19).

## Author Contributions

All authors contributed to the writing of the manuscript. MN and AF carried out the bulk of the experimental work. JH and A-TE carried out the mouse infection work. QZ carried out parts of the bioinformatic analyses. UK supervised the experimental work and provided the conceptual framework for the project. AM provided the input into the conceptual framework of the work and contributed to student supervision.

## Conflict of Interest

The authors declare that the research was conducted in the absence of any commercial or financial relationships that could be construed as a potential conflict of interest.

## Footnotes

1 http://www.prodoric.de/vfp/, accessed 28/12/2020
