# Supplementary material for: The Alternative Sigma Factor RpoE2 Is Involved in the Stress Response to Hypochlorite and in vivo Survival of Haemophilus influenzae
Source: Front Microbiol. 2021 Feb 12;12:637213. doi: 10.3389/fmicb.2021.637213 (PMC7907618; doi:10.3389/fmicb.2021.637213)
Supplement: Supplementary file 1 [file Data_Sheet_1.docx]

The alternative sigma factor RpoE2 is involved in the stress response to hypochlorite and *in vivo* survival of *Haemophilus influenzae*

Marufa Nasreen^1†^, Aidan Fletcher^1†^, Jennifer Hosmer^1^, Qifeng Zhong^1^, Ama-Tawiah Essilfie^2^, Alastair G. McEwan^1^, Ulrike Kappler^1*^

^1^Australian Infectious Diseases Research Centre, School of Chemistry and Molecular Biosciences, The University of Queensland, St. Lucia, Qld 4072, Australia

^2^QIMR Berghofer Medical Research Institute, 300 Herston Road, Herston QLD 4006, Australia

Supplementary Data

**Table S1 – Bacterial strains and plasmids used in this study**

| ***Bacterial Strain/Plasmid*** | ***Description*** | ***Reference*** |
| --- | --- | --- |
| **Bacterial Strains** |  |  |
| *E. coli* DH5α | F–, Φ80lacZΔM15, Δ(lacZYA-argF), U169, recA1,  endA1, hsdR17 (rK–, mK+), phoA, supE44, λ–, thi1, gyrA96 and relA | Invitrogen |
| *E.coli* Jm109λpir | endA1, recA1, gyrA96, thi, hsdR17 (rk–, mk+), relA1, supE44, Δ(lac-proAB), F´ traD36, proAB, laqIqZΔM15, lysogenized with λpir bacteriophage | Promega |
| Hi2019 | Wild-type non-typeable *H. influenzae Strain* 2019, COPD isolate | Campagnari, et al. ^1^ |
| Hi2019^Δ^*^rpoE2^* | *H. influenzae* Strain 2019, rpoE2::Kan | This Study |
| **Protein Expression Plasmids** | | |
| pProexHtb | Protein expression plasmid with a *trc* promoter. Confers N-terminal 6x His tag. Amp^R^. | Invitrogen |
| pProex_Hi- RpoE2 | Protein expression plasmid with the Hi2019 *rpoE2* gene inserted in the cloning site. N-terminal 6x His tag. Amp^R^. | This study |
| pProex_Hi- RpoE2-ASF | Protein expression plasmid with the Hi2019 *rpoE2* and *hrsE* genes inserted in the cloning site. N-terminal 6x His tag. Amp^R^. | This study |
| **Promoter Analysis Plasmids** | | |
| pMU2385 | Promoter fusion plasmid. Contains lacZ gene with no promoter. Tp^R^. | Praszkier, et al. ^2^ |
| pMU2385_Hi*msrAB* | Promoter fusion plasmid with the promoter for the *msrAB* gene fused to the *lacZ* gene. Tp^R^. | This study |
| pMU2385_Hi*dmt* | Promoter fusion plasmid with the promoter for the *dmt* gene fused to the *lacZ* gene. Tp^R^. | This study |
| pMU2385_Hi*rpoE2_*284 | Promoter fusion plasmid with the 284bp promoter region for the *rpoE2* gene fused to the *lacZ* gene. Tp^R^. | This study |
| pMU2385_Hi*rpoE2_*865 | Promoter fusion plasmid with the 865bp promoter region for the *rpoE2* gene fused to the *lacZ* gene. Tp^R^. | This study |
| **Plasmids used to construct a Hi2019^Δ^*^rpoE2^* strain** | | |
| pBlue_HirpoE2 | Standard cloning vector with Hi2019 *rpoE2* gene inserted in the MCS. Amp^R^. | This study |
| pBlue_Hi*rpoE*2_IS_kan | Standard cloning vector with Hi2019 *rpoE2* gene inserted in the MCS. A Kanamycin resistance cassette from pUC4K is inserted within the *rpoE2* gene. Amp^R^ Kan^R^. | This study |

1. Campagnari, A. A.; Gupta, M. R.; Dudas, K. C.; Murphy, T. F.; Apicella, M. A., Antigenic diversity of lipooligosaccharides of nontypable *Haemophilus influenzae*. *Infect. Immun.* **1987,** *55* (4), 882-7.

2. Praszkier, J.; Wilson, I. W.; Pittard, A. J., Mutations affecting translational coupling between the *rep* genes of an IncB miniplasmid. *J. Bacteriol.* **1992,** *174* (7), 2376-2383.

**Table S2:** oligonucleotide primers used in this study

| **Primer Name** | **Primer Sequence** |
| --- | --- |
| **Construction of reporter gene plasmids** |  |
| Hi2019msrABpr BamH F | AAAAGGATCCCGCTGATACCATAAAACTAA |
| Hi2019msrABpr Eco R | AAAAGAATTCTGTTTTTGATAGTTTCATTGCT |
| Hi2019dmtpr BamH F | AAAAGGATCCGATAGTTTCATTGCTTTTCCT |
| Hi2019dmtpr Eco R | AAAAGAATTCCGCTGATACCATAAAACTAA |
| Hi2019rpoE2 prom Eco F | AAAAGAATTCAACATTCATTTTAATTCCTTTAA |
| Hi2019rpoE2 prom280 Bam R | AAAAGGATCCAAGTCGTTGGAAAAGCGAG |
| Hi2019rpoE2 prom800 Bam R | AAAAGGATCCTGAATTTTCCTTTCTACGAACT |
|  |  |
| **Protein expression plasmid construction** |  |
| HI2019_pPro_rpoE2_BamHF | AAAAGGATCCATGAATGTTATTTCTGATCTT |
| HI2019_pPro_rpoE2_XbaI R | AAAATCTAGACTAAAGTTTTTTTGAAAGACAGT |
| HI_rpoE2AS_pProEcoR | AAAAGAATTCCATTACTCCTTTTGCTTTCAC |
| pProex_seq_fwd | TGGCAAATATTCTGAAATGAG |
|  |  |
| **RpoE2 gene knockout plasmid** |  |
| HI_RpoE2ko_upF_Eco | AAAAGAATTCTTTGTCAAAACCGCTTCTCAAACAA |
| HI_RpoE2ko_upR_BamH | AAAAGGATCCAAGGGCGGTTTGTTTTAGAAAGTGA |
| HI_RpoE2ko_downF_BamH | AAAAGGATCCTCACTTTCTAAAACAAACCGCCCTT |
| HI_RpoE2ko_downR_SacI | AAAAGAGCTCAAATTTGCACTGTAAAGCGCACG |
| pUC4K_PCR F | GTTGGGTAACGCCAGGGTTTTCC |
| pUC4K_PCR R | TCCGGCTCGTATGTTGTGTGGAA |
|  |  |
| **qPCR primer** |  |
| HiallQP_16SF | GGGGTAGAATTCCACGTGTAGCGG |
| HiallQP_16SR | TCCTGTTTGCTCCCCACGCTT |
| Hi QP gyrA F | TTGGGCGTGCATTACCTGACGTT |
| Hi QP gyrA R | CCCACAACACGCGCTGATTTTAC |
| HI_QP_rpoE2F | TTGGCGGGTAAACAAGTCAAACAAG |
| HI_QP_rpoE2R | TGAAAAGGGGCATTGGAAACC |
| Hi QP msrAB F | ACCAACAAGGTGCGGATAAAGGC |
| Hi QP msrAB R | GCGGCTCTGCATATTGTGCTTGT |
| Hi QP mtsZ F | AACAAACGGGTTACCACCTGC |
| Hi QP mtsZ R | CCATTAGCGCGTATTGCTGAT |
| Hi QP sodA F | CGTTCAATCGCATCTTTTAATGCGC |
| Hi QP sodA R | TGCAGAAAAACGTGGAGCATTACGT |
| Hi QP htkE F | CCGTCAAATTCCAGTAAACCGTCC |
| Hi QP htkE R | TGGCTGAAGCTGTTTGGCTCGTAGT |
| Hi_dps_QP_F | TCGGACTAGATAAAGTTCAATCAGCAGAATT |
| Hi_dps_QP_R | AGTTTACGCCTTTAATGTTCCAGTGGTAGC |
| Hi_8420DMT_QP_F | TTAATCGGTTTTCTTGGCGTTTCCA |
| Hi_8420DMT_QP_R | GCCAATAAATGCCATTCCCGCTA |
| Hi QP pdgX F | GCTTGTGAAAAACGGCGTAGTTGAA |
| Hi QP pdgX R | TGCACTTGGTGTTGTGGTGCAAGGT |
| Hi7455_QP_F | GCAGGATTAGAAAAATGGAATGGACAAA |
| Hi7455_QP_R | TAATTCTGATCCCATTGCAACGTGC |
| Hi7460_QP_F | TTAGCAAGTGCATTAACGATGGCAGTAG |
| Hi7460_QP_R | TGCGGCTTTCGTTTTGACACATTTA |
| Hi7465_QP_F | ATTAGGTTATCGTCGGAATTTAGCTGAGG |
| Hi7465_QP_R | TGATAACGAGCCATTCCACCCATTT |
| Hi7470_QP_F | GCAGATCCTTTAAATGGTTATGCTGCAA |
| Hi7470_QP_R | TGGCTCAATATGTAATGGTGCTTCAACA |

**Table S3: Strains used in reporter gene assays**

| ***Strain*** | ***Description*** | ***Reference*** |
| --- | --- | --- |
| pMU-/pPro- | *E.coli* Jm109λpir with pMU2385 and pProexHtb plasmids | This study |
| pMU-/pProRpoE2 | *E.coli* Jm109λpir with pMU2385 and pProex_Hi2019- RpoE2 plasmids | This study |
| pMU-/pProRpoE2+AS | *E.coli* Jm109λpir with pMU2385 and pProex_Hi2019- RpoE2-ASF plasmids | This study |
| pMUmsrAB/pPro- | *E.coli* Jm109λpir with pMU2385_Hi*msrAB* and pProexHtb plasmids | This study |
| pMUmsrAB/pProRpoE2 | *E.coli* Jm109λpir with pMU2385_Hi*msrAB* and pProex_Hi2019- RpoE2 | This study |
| pMUmsrAB/pProRpoE2+AS | *E.coli* Jm109λpir with pMU2385_Hi*msrAB* and pProex_Hi2019- RpoE2-ASF plasmids | This study |
| pMUrpoE2_284/pPro- | *E.coli* Jm109λpir with pMU2385_Hi*rpoE2_*284 and pProexHtb plasmids | This study |
| pMUrpoE2_284/pProRpoE2 | *E.coli* Jm109λpir with pMU2385_Hi*rpoE2_*284 and pProex_Hi2019- RpoE2 plasmids | This study |
| pMurpoE2_284/pProRpoE2+AS | *E.coli* Jm109λpir with pMU2385_Hi*rpoE2_*284 and pProex_Hi2019- RpoE2-ASF plasmids | This study |
| pMUrpoE2_865/pPro- | *E.coli* Jm109λpir with pMU2385_Hi*rpoE2_*865 and pProexHtb plasmids | This study |
| pMUrpoE2_865/pProRpoE2 | *E.coli* Jm109λpir with pMU2385_Hi*rpoE2_*865 and pProex_Hi2019- RpoE2 plasmids | This study |
| pMurpoE2_865/pProRpoE2+AS | *E.coli* Jm109λpir with pMU2385_Hi*rpoE2_*865 and pProex_Hi2019- RpoE2-ASF plasmids | This study |

**Table S4:** MS/MS analysis of recombinant Hi RpoE2 co-purified with the HrsE Hi RpoE2 ASF.

RpoE2 and the HrsE ASF are shown in orange, subunits of the RNA polymerase core enzyme that might also interact with RpoE2 are shown in yellow. Please note that the database searches wer conducted against a *Haemophilus influenzae* strain 2019 database, but as HiRpoE2 and HrsE were expressed in *E. coli*, all other proteins identified are likely to be co-purifying *E. coli* proteins rather than actual *Haemophilus influenzae* proteins.

N – protein number, total score – protein score, a score >4 indicates identification at >99% confidence, % Cov(95) – percent sequence coverage with at least 95% confidence. Peptides (95%) – no of peptides identified with at least 95% confidence. Accession number – NCBI protein accession number, Name – annotation in the Hi2019 genome.

**Figure S1** – Activity of pMU2385 lacZ gene in the presence of pProex Htb, pProex – RpoE2 (pProRpoE2), and pProex RpoEs HrsE antisigmafactor (pProRpoE2+AS). These are control reactions that indicate the baseline activity of the promoterless *lacZ* gene in the presence of RpoE2 or RpoE2 and HrsE.


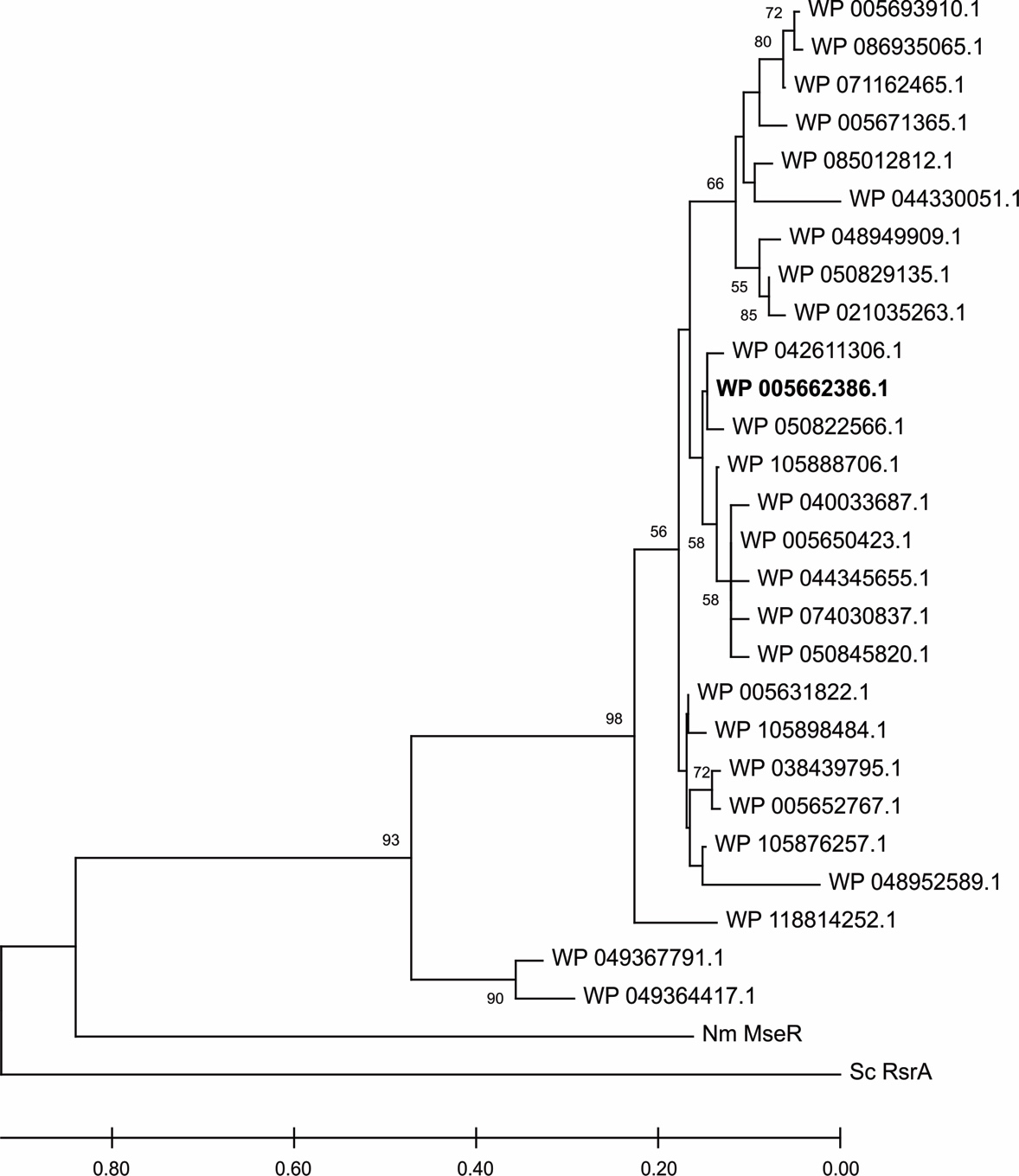


**Figure S2** – Phylogenetic relationships between *H. influenzae* HrsE sequence variants. The phylogenetic tree was created using the Neighbor-joining method with robustness testing using 500 bootstrap cycles. The majority of Hi HrsE protein sequences formed two related clades while the remaining 3 sequence types (WP_049364417, WP_049367791, WP_048952589) are more divergent sequences. The Hi2019 RpoE2 belongs to the WP_005662386.1 sequence type (bold). ASF sequences from *Neisseria meningitidis* Nm3682 (‘Nm MseR’, AIZ23067.1) and *Streptomyces coelicolor* A3(2) RsrA (‘Sc RsrA’; CAB94602.1) were used as outgroups.

**Figure S3:** ClustalW2 alignment of Hi HrsE, *Neisseria meningitidis* MseR (Nm MseR) and *Streptomyces coelicolor* RsrA (Sc RsrA) ASF sequences. Bold = amino acids of the ZAS zinc -binding motif, ***** amino acids conserved in all aligned sequences, **:** substitution with amino acids with very similar chemical properties, **.** substitution with amino acids that have weakly related chemical properties.

**Figure S4** – Resistance of Hi2019^WT^ and Hi2019^Δ^*^rpoE2^* to paraquat. No significant differences in cell numbers were detected using 2-Way-ANOVA (Strain/paraquat concentration).

**Figure S5** – Resistance of Hi2019^WT^ and Hi2019^Δ^*^rpoE2^* to polymyxin **(A)** and human normal serum **(B)**. No significant differences in cell numbers were detected using 2-Way-ANOVA (Strain/polymyxin(serum) concentration).
